# Systematic Review and Meta-Analyses of The Interaction Between HIV Infection And COVID-19: Two Years’ Evidence Summary

**DOI:** 10.3389/fimmu.2022.864838

**Published:** 2022-05-10

**Authors:** Yehua Wang, Yewei Xie, Siyue Hu, Wei Ai, Yusha Tao, Huilin Tang, Fengshi Jing, Weiming Tang

**Affiliations:** ^1^ College of Pharmacy, University of Florida, Gainesville, FL, United States; ^2^ Duke Global Health Institute, Duke University, Durham, NC, United States; ^3^ Institute for Healthcare Artificial Intelligence, Guangdong Second Provincial General Hospital, Guangzhou, China; ^4^ University of North Carolina at Chapel Hill Project-China, Guangzhou, China; ^5^ School of Public Health, Nanjing Medical University, Nanjing, China; ^6^ Institute for Global Health and Infectious Diseases, School of Medicine, The University of North Carolina at Chapel Hill, NC, United States

**Keywords:** human immunodeficiency virus (HIV), Coronavirus-19 (COVID-19), SARS-CoV-2, systematic review, meta-analysis

## Abstract

**Introduction:**

During the COVID-19 pandemic, people living with HIV (PLWH) were considered to be at risk of worse COVID-19 outcomes once infected. However, the existing evidence is inconsistent. This systematic review and meta-analysis aimed to compare the risk of SARS-CoV-2 infection, severe COVID-19 symptoms, and mortality among PLWH and patients without HIV.

**Method:**

The articles included studies published in PubMed, Medline, Embase, and Cochrane between December 1, 2019, and December 1, 2021. We included the original studies published in English focusing on observational studies assessing the risk of SARS-CoV-2 infection, severe COVID-19 symptoms, and mortality among PLWH. Four independent reviewers extracted data. STrengthening the Reporting of OBservational studies in Epidemiology-Modified (STROBE-M) checklist was used for quality assessment. For the results with heterogeneity I^2^ >75%, a random-effects model was employed. Otherwise, a fixed-effects model was used. The risk of SARS-CoV-2 infection, severe COVID-19 symptoms, and mortality were compared with and without HIV.

**Results:**

We included a total of 32 studies and 71,779,737 study samples, of whom 797,564 (1.11%) were PLWH. Compared with COVID-19 patients without HIV infection, PLWH had comparable risk of SARS-CoV-2 infection (adjusted Risk Ratio=1.07, 95% CI: 0.53-2.16, I^2 =^ 96%, study n=6, n=20,199,805) and risk of developing severe COVID-19 symptoms (aRR=1.06, 95% CI: 0.97-1.16, I^2 =^ 75%, n=10, n=2,243,370). PLWH, if infected with SARS-CoV-2, were found to have an increased risk of mortality compared with people without HIV (aRR=1.30, 95% CI: 1.09-1.56, I^2 =^ 76%, study n=16, n=71,032,659). This finding was consistent across different subgroup analyses.

**Conclusion:**

PLWH are at increased risk of COVID-19 related mortality once infected. The local health system should, on the one hand, strengthen COVID-19 prevention and clinical management among PLWH to avoid infection and, on the other hand, sustain the HIV care continuum for PLWH for HIV management.

## 1 Introduction

Every minute, around 190 new Coronavirus Disease 2019 (COVID-19) cases are reported, and three individuals die from COVID-19 throughout the globe. The outbreak of COVID-19, caused by the severe acute respiratory syndrome coronavirus 2 (SARS-CoV-2), has resulted in one of the most devastating pandemics in human history (1). By January 20, 2021, it has caused over 3 billion illnesses and approximately 5.5 million deaths (2). As a result of the availability of the COVID-19 vaccine and a better understanding of the COVID-19 treatment, the overall COVID-19 mortality is decreasing. However, the immunocompromised populations are still at an increased risk of mortality. Due to abnormal humoral and cellular immunity, people living with HIV (PLWH) were considered to be at an increased risk of severe Covid-19 outcomes once infected (3, 4). On the other hand, PLWH of long-term use of ART also has a higher chance of suffering from different chronic conditions such as hypertension than HIV negative population, which as a result, worsen their outcome once infected by SARS-CoV-2 (4).

Considering that there are approximately 38 million PLWH globally and the potential increased risk of severe COVID-19 and mortality for this population, it is warranted to systematically assess the current vulnerability of PLWH SARS-CoV-2 infection and related outcomes (5). However, the recent epidemiological evidence on PLWH infected with SARS-CoV-2 has been inconsistent (6). Although there have been meta-analyses assessing the impact of HIV infection on COVID-19 outcomes among PLWH, the time frame of evidence inclusion was early, before January 2021, which did not cover cases with new variants (e.g., delta variant) (7, 8). After that, several variants have emerged and changed the global COVID-19 pandemic significantly. Meanwhile, more studies with larger sample sizes, more sophisticated study designs, and broader geographic coverage. It is high time we looked back for two years to summarize and elucidate how COVID-19 infection differs between PLWH and people without HIV infection. By filling up this knowledge gap, it will better characterize PLWH’s vulnerability during this COVID-19 pandemic and thus advocate for more attention and care for this population to achieve better health equity.

This systematic review and meta-analysis aimed to compare the risk of SARS-CoV-2 infection, severe COVID-19 symptoms, and death of PLWH with those without HIV.

## 2 Methods

### 2.1 Information Source and Search Strategy

This systematic review and meta-analysis were conducted and reported per Preferred Reporting Items for Systematic Reviews and Meta-Analyses (PRISMA) statement (9). We searched PubMed, Medline, Embase, and Cochrane for publications from December 1^st^, 2019 to December 1^st^, 2021 using the combination of HIV/AIDS-related terms (“HIV”, “human immunodeficiency virus*”, “AIDS”, “acquired immunodeficiency syndrome”) and COVID-19 related terms (“COVID-19”, “SARS-CoV*”, “2019-nCoV”, “nCoV” and “novel coronavirus”). We also included terms related to observational studies such as “cohort”, “cross-sectional”, and “case-control” (See **Supplementary Table 1** for detailed search terms).

### 2.2 Eligibility Criteria

For eligible studies, we included original observational studies published in English. The included studies should consist of both HIV positive and HIV negative COVID-19 cases in the study population. The outcomes of interests should at least contain one of the following three outcomes 1) the risk of SARS-CoV-2 infection, 2) the risk of developing severe COVID-19 symptoms, and 3) the risk of COVID-19 related mortality. We hereby defined severe COVID-19 symptoms according to the WHO COVID-19 clinical management guidelines (10). This study’s main objective is to pool and compare the outcomes between PLWH and people without HIV infection. On the other hand, due to age, sex, and comorbidities’ impact on the COVID-19 treatment outcomes, we required the included studies to have results adjusted for potential confounders. Studies without confounder adjustment were not included. Original studies focusing on molecular, genetics, and sociological perspectives without the above outcomes of interest were excluded. Studies without primary data, such as comments and reviews, were excluded. Case reports and case series were also excluded.

### 2.3 Literature Screening and Data Extraction

Two groups of reviewers (YW, YX, WT and SH, WA, YT) independently screened the literature titles, abstracts, and full-texts and identified eligible literature for data extraction. The two reviewers discussed any disagreement. A third reviewer (WT) was consulted for reconciliation if an agreement was not reached.

The following variables were extracted from the final included studies: the first author, published year, country, study design, study settings, study periods, sample size, diagnosis methods, patient-basic information (e.g., mean age and sex), HIV-related information [e.g., latest CD4 count, latest HIV viral load and whether current receiving antiviral therapy (ART)], comorbidities, and COVID-19 related outcomes (infection, severe COVID-19 symptoms, and morality). If reported, we extracted both the crude and adjusted estimation from the included studies. The adjusted results with the maximum number of adjustments were selected (11).

### 2.4 Analysis and Synthesis

To synthesize the impact of the HIV/SARS-CoV-2 co-infection on the three outcomes, we conducted pooled analyses based on the gathered effect estimates and standard errors. Pooling was accomplished using the inverse variance method. Considering the variation in the types of effect estimation, including odds ratio (OR), risk ratio (RR), and hazard ratio (HR), and for ease of interpretation, we converted these estimations into a standard metric, risk ratio (12, 13). The pool results were expressed as RR, with 95% confidence intervals, I^2^, the heterogeneity metric, number of studies pooled, and number of sample sizes included. The heterogeneity between the studies in the comparison was assessed by calculating I^2^. A fix-effects model would be employed for the results with I^2^ ≤75%. A random-effects model would be employed for the results with I^2^ >75% (14). Statistical significance was based on a p-value of less than 0.05.

### 2.5 Subgroup Analysis

Considering the diversities in the healthcare system and variations in the study setting and their potential impact on the pooling, we conducted subgroup analyses based on World Bank income classification (high-income (HIC) vs. low-middle-income countries (LMIC)), studying inpatients cases only or both inpatient and outpatient cases, single-center or multi-center study. If an investigation could not be classified into either subgroup, it was excluded from the subgroup analysis. Adjusted RRs were used in the subgroup analysis.

### 2.6 Quality and Publication Bias Assessment

Two reviewers (SH and WA) independently assessed the quality of the included studies. The STrengthening, the Reporting of OBservational studies in Epidemiology-Modified (STROBE-M) checklist, was used for the quality assessment (15, 16). We calculated the adherence score based on the checklist results. The studies scored ≥ 85 would be ranked as “Excellent” in quality. The studies scored between 70 to 85 would be ranked as “Good”. The studies scored between 50 to 70 would be ranked as “Fair”. Those studies scored below 50 would be ranked as “Poor” (16). We generated funnel plots that utilized Eggers’ test to examine small sample effects as a possible indicator of publication bias (p<0.05) in our meta-analysis models to assess the potential publication bias. Articles were downloaded and managed by Endnote X9. Data were extracted and entered into Microsoft Excel (2016). The meta-analyses were performed by the R v4.02 and R Studio (2020), using the R package *meta*. Quality assessment was performed by using the STROBE-M checklist.

## 3 Results

### 3.1 Overview

**Figure 1:**
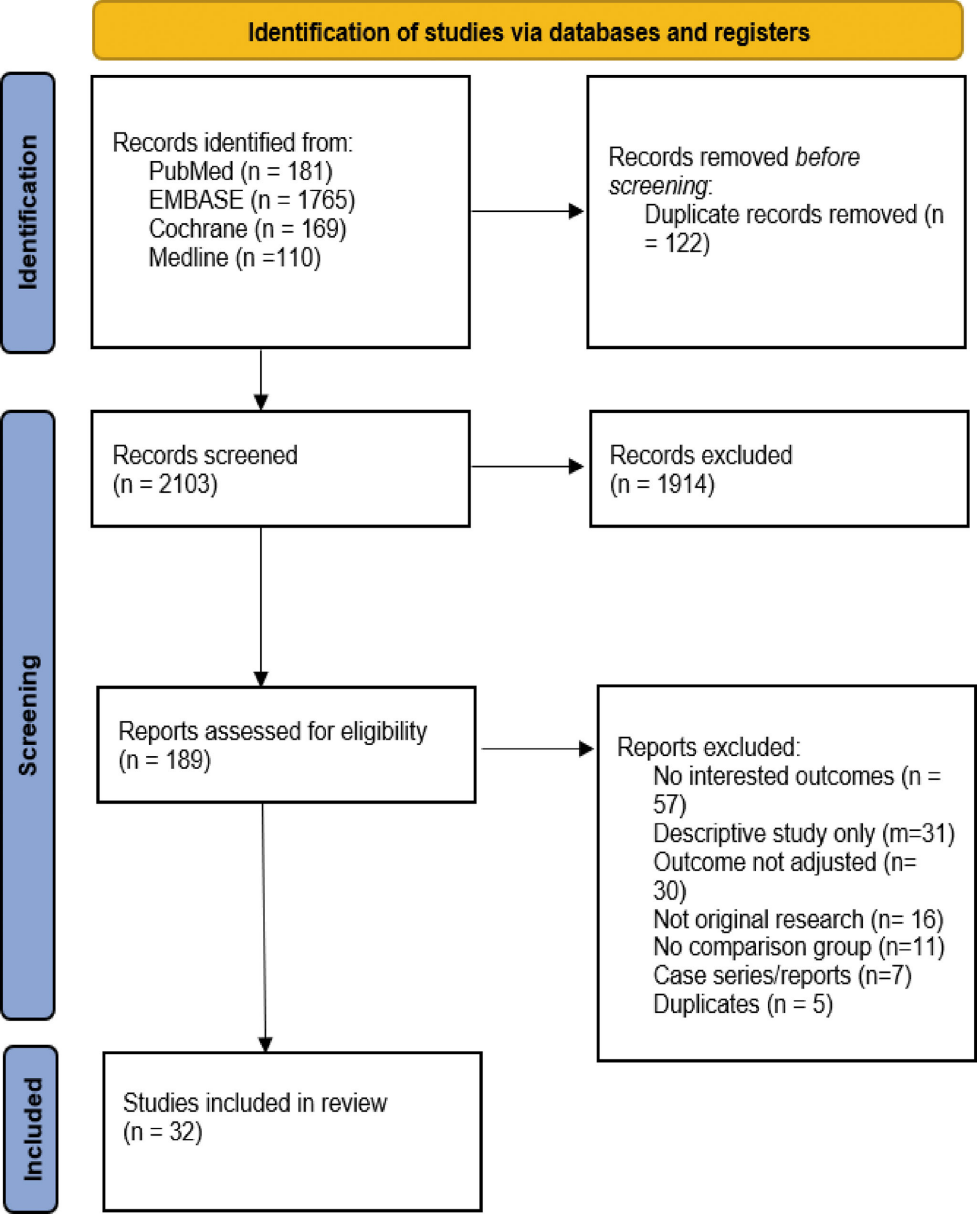
Preferred Reporting Items for Systematic Reviews and Meta-analysis (PRISMA) chart for the literature search.

A total of 2,225 articles were identified from the databases. Overall, 122 of them were detected to be duplicates by Endnote, leaving 2103 articles for the initial title & abstract screening. Based on the abstract and title screening, 1,914 articles were excluded. Among the 189 articles screened for full text, an additional 157 articles were excluded, leaving the rest 32 articles included for the analyses (**Figure 1**) (17–48).

### 3.2 Study Description

The analysis included a total of 71,779,737 sample size, of whom 797,564 (1.11%) were PLWH. These studies were conducted in 10 countries and represented North America (n=14), Africa (n=7), Europe (n=10), and Asia (n=1). 17 (53.12%) of the studies were conducted in hospital settings, and 15 of them were conducted in other settings such as registries, surveillance system databases, national cohorts. 25 (78.12%) of the included studies were multi-center studies, and 13 (40.62%) of these studies specifically focused on hospitalized COVID-19 cases. The study information was summarized in **Supplementary Table 2**.

**Supplementary Table 3** summarized patient-level sociodemographic variables as well as clinical information of the patients. 58% of the study population were male. The mean age of PLWH was 52.12 (Standard deviation (SD): 6.82), while the mean age of patients without HIV was 55.34 (SD: 5.95). Among PLWH, on average, 91% of them were on ART (range: 70% - 100%). In terms of HIV viral suppression rate, on average, 77% of the studied population had achieved HIV viral suppression (range: 31%-97%). About 87% (92,714/106,568) of the PLWH had at least one comorbidity besides SARS-CoV-2 infection, which was higher than HIV- patients [79% (37,838/47,746)]. Hypertension, diabetes, and obesity were the top three most reported comorbidities among PLWH.

### 3.3 Meta-Analyses

**Figure 2:**
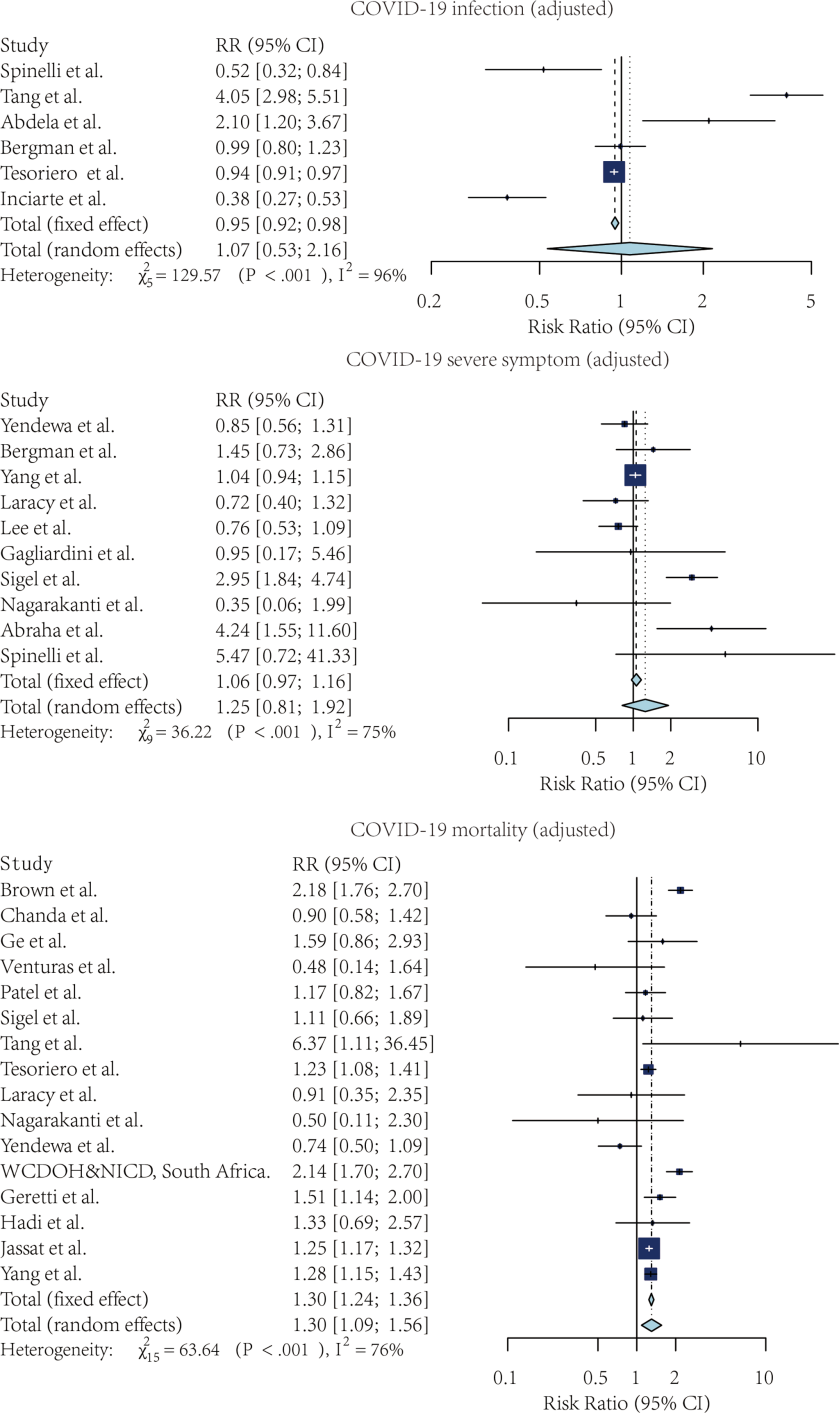
The forest plot of the crude risk ratio of SARS-CoV-2 infection, Severe COVID-19 symptoms, and COVID-19 mortality, comparing the patients with and without HIV.

**Figure 3:**
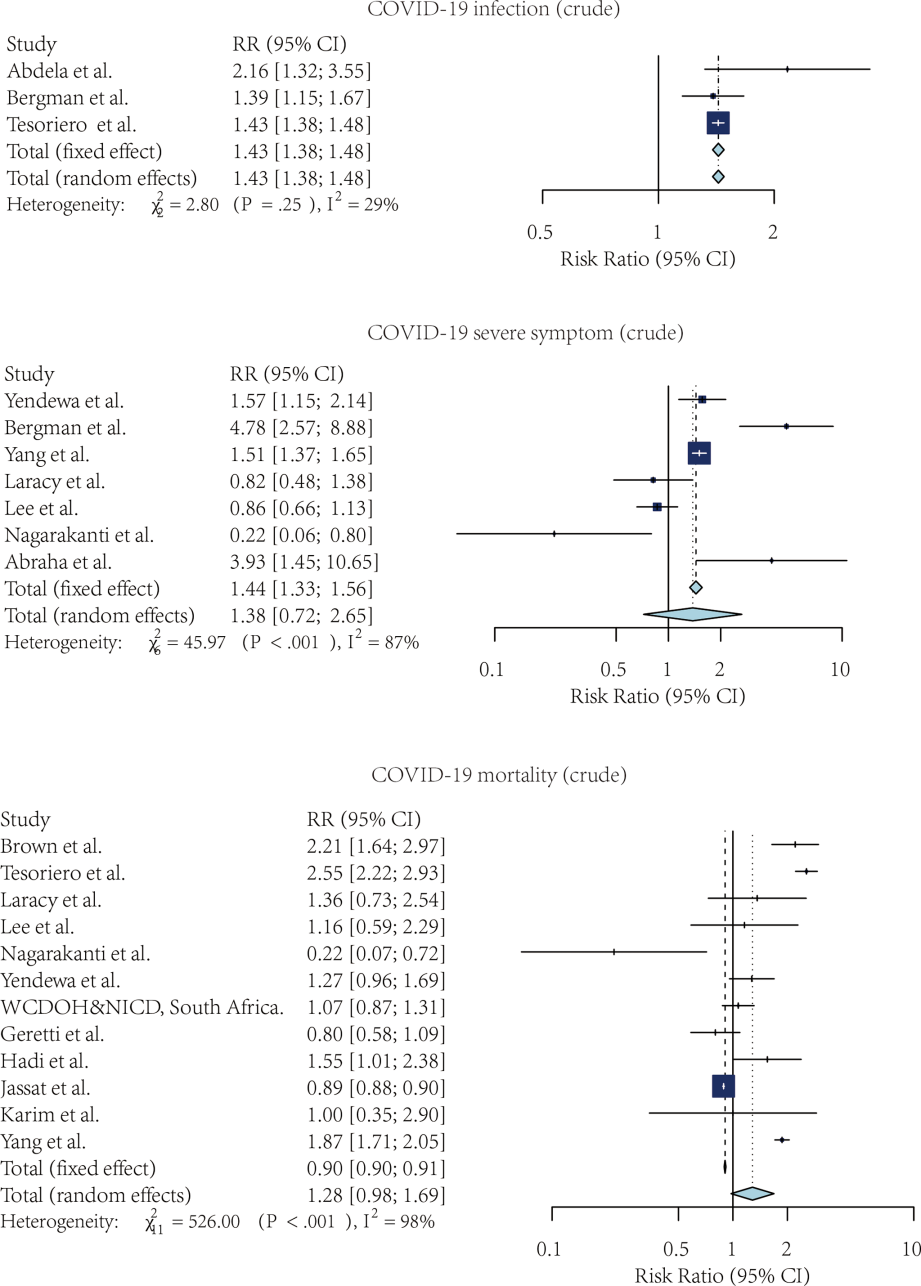
The forest plot of the adjusted risk ratio of SARS-CoV-2 infection, Severe COVID-19 symptoms, and COVID-19 mortality, comparing the patients with and without HIV.

**Figures 2**, **3** showed the forest plots for the three meta-analyses related to the crude and adjusted risk of SARS-CoV-2 infection, severe COVID-19 symptoms, and COVID-19 mortality.

#### 3.3.1 Risk of SARS-CoV-2 Infection

Four studies compared the risk of SARS-CoV-2 infection without adjustment. According to the pooled crude risk ratio, there was an increased risk of SARS-CoV-2 infection among PLWH compared with people without HIV infection (RR=1.43, 95% CI: 1.38-1.48, I^2^ = 29%, study n=3, n=19,956,496). Eight studies provided an adjusted risk of SARS-CoV-2 infection estimation. After the adjustment, the susceptibility to SARS-CoV-2 infection was comparable between the two groups (aRR=1.07, 95% CI: 0.53-2.16, I^2^ = 96%, study n=6, n=20,199,805).

#### 3.3.2 Risk of Severe COVID-19 Symptoms

Nine studies compared the risk of developing severe COVID-19 symptoms between HIV-positive and negative groups. PLWH had a higher risk of developing severe SARS-CoV-2 symptoms once infected. This effect was marginally significant for both crude estimation (RR=1.38, 95% CI: 0.72-2.65, I2 = 87%, study n=7, n=2,239,955) and adjusted estimation (aRR=1.06, 95% CI: 0.97-1.16, I2 = 75%, n=10, n=2,243,370).

#### 3.3.3 Risk of Mortality Among COVID-19 Patients

PLWH infected with COVID-19 also had a higher mortality risk than people without HIV infection. Overall, 13 studies provided crude estimations. The pooled crude risk ratio of mortality was 1.28 (RR=1.28, 95% CI: 0.98-1.69, I^2^ = 98%, study n=12, n=87,907,007), which was marginally significant. Nineteen studies had an adjusted mortality risk ratio. After adjustment, PLWH had a statistically significant higher risk of mortality than those not infected with HIV (aRR=1.30, 95% CI: 1.09-1.56, I^2^ = 76%, study n=16, n=71,032,659).

### 3.4 Comparison of Unadjusted RRs With Adjusted RRs

We compared the pooled crude estimates with the pooled adjusted estimates. The estimation was reduced as the estimates were adjusted for the potential confounders, including age, sex, and comorbidities, suggesting the importance of controlling for confounding minimizes the strength of association.

#### 3.4.1 Subgroup Analysis

We hereby presented mortality, one of the most critical COVID-19 related outcomes, using adjusted RR (supplement 2: forest plots of subgroup analysis).

In high-income countries (HIC), the adjusted risk ratio was 1.33 (aRR=1.33, 95% CI:1.24-1.42, I^2^ = 71%, study n=12). In low- and middle-income countries (LMIC), the adjusted risk ratio was 1.22 (aRR=1.22, 95% CI: 0.74-2.00, I^2^ = 88%, study n=4).

Regarding the study population, we divided the studies into 1) only included inpatient cases and 2) included both inpatient and outpatient cases. For the studies with only hospitalized patients, the pool adjusted risk ratio of mortality was 1.25 (aRR=1.25, 95% CI:1.18-1.32, I^2 ^= 14%, study n=6). For the studies with inpatient and outpatient cases, the pool adjusted risk ratio of mortality was 1.38 (aRR=1.38, 95% CI:1.06-1.79, I^2^ = 83%, study n=10). The findings were more consistent among inpatient studies.

We divided the studies into multi-center studies versus single-center studies regarding the study settings. We found a 44% increase in RR of mortality among PLWH compared with patients not infected with HIV (aRR=1.34, 95% CI:1.12-1.60, I^2^ = 78%, study n=14). On the other hand, the difference in mortality risk was not significant among single-center studies (aRR=0.49, 95% CI:0.19-1.27, I^2^ = 0%, study n=2).

### 3.5 Study Quality and Publication Bias Assessment

The mean STROBE-M adherence score (%) was 69.1% (SD: 11.2, range: 40.5-88.1). Overall, the included studies’ quality is “Fair” (**Supplementary Table 4**). Two studies were of excellent quality, 15 were of good quality,13 were of fair quality, and two were of poor quality. Visual inspection of the funnel plot did not show obvious asymmetry. Egger’s test for asymmetry was insignificant (p=0.80). We could not conclude there was a significant publication bias among the included studies. The funnel plot was shown in (supplement 3: funnel plot).

## 4 Discussion

Given COVID-19 is very likely to become a long-lasting infectious disease (49). Thus, understanding its interaction with other infectious diseases, such as HIV, will provide us with crucial messages for planning future measures. This was a two-year summary of the evidence on HIV and SARS-CoV-2 infection interactions. Compared with our last systematic review in early 2021 (8), there were more multi-center studies, more analyses with statistical adjustments, and more studies from different countries, both from HICs and LMICs.

We identified an increased risk of COVID-19 related mortality among PLWH compared with patients without HIV infection. Considering the role of age and comorbidities in impacting the COVID-19 infection outcome, after the adjustment, there was still an increased risk of mortality among PLWH, indicating HIV infection was possibly an independent risk factor of death caused by COVID-19. The subgroup analyses further confirmed the findings across LMICs and HICs, inpatient-only populations, and inpatient and outpatient mixed populations. This finding indicates the importance of COVID-19 prevention among PLWH and more careful management when PLWH presented with COVID-19.

We did not identify an increased risk of SARS-CoV-2 infection among PLWH. This was possibly due to public health prevention measures such as quarantine and shutdowns. Also, it was possible that PLWH, knowing their HIV infection, had a stronger awareness of COVID-19 prevention and avoided congregation. At the same time, the global-wide row out of the COVID-19 vaccine campaign also plays an indispensable role in stopping the spread.

Regarding the SARS-CoV-2 infection severity among PLWH, we did not identify a statistically significant increased risk of developing severe symptoms for PLWH. A few reasons may have led to this phenomenon. From an immunology perspective, since CD4 T cells play a pivotal role in innate and cellular immunity, the dysregulation of CD4 T cell activities and functions in PLWH could increase the risk of having worse outcomes once infected with infected SARS-CoV-2 compared with those not infected with HIV. One explanation could be due to the high viral suppression rate and ART uptake rate, which were important influencers of PLWH’s vulnerability to other infections. Since the majority of the PLWH had a controlled HIV status, this study was unable to assess the COVID-19 outcomes among patients with uncontrolled HIV due to limited study numbers and sample size. However, there has been evidence that patients with uncontrolled HIV would be more likely to develop severe Covid-19 symptoms, which aligned with the immunology mechanism (50, 51). On the other hand, the insignificant results could be due to the high heterogeneity of the included analyses, which led to a wider confidence interval estimation. Considering these facts, future studies and meta-analyses on how the outcome of SARS-CoV-2 infection are moderated by ART status, CD4 levels, COVID-19 vaccination status, and HIV viral load are warranted.

To our knowledge, this is the most recent review that covers the longest period since the detection of COVID-19 in December 2019. This review collected the relevant studies around the globe for a more comprehensive assessment of the SARS-CoV-2/HIV co-infection. In recognition of the impact of different potential confounders, we pooled the impact of crude interesting outcomes and the outcomes adjusted for potential confounders. We also conducted a series of subgroup analyses of mortality to assess the robustness of our findings.

Our studies have several limitations. Our studies could not assure if all SARS-CoV-2 infection outcomes were clinically confirmed since most of the studies confirmed the case based on RT-PCR only. There were possibilities of misclassification. Second, we were not able to analyze the outcomes of patients with poor HIV control compared with those without HIV infection due to the insufficiency of the evidence. However, a study has shown that patients with uncontrolled HIV are at risk of developing severe COVID-19 (50, 52). Third, the identified studies did not distinguish different SARS-CoV-2 variants with various contingencies and mortality rates (53).

Our study has several implications. From the policy perspective, the policymakers and healthcare system should reinforce the COVID-19 prevention among PLWH and maintain the HIV care continuum during the COVID-19 pandemic to ensure PLWH are at good HIV control (54–56). Also, it is important to advocate for the rolling out of the COVID-19 vaccine among PLWH to reduce the risk of developing severe COVID-19 and death among PLWH once infected (17), especially in countries with a high HIV burden. In terms of research perspective, first, future studies should focus more on evaluating the role of HIV treatment status, especially poor HIV control, its impact on SARS-CoV-2 infection outcomes. Second, considering the ever-mutating nature of SARS-CoV-2, it is necessary to assess different variants’ interactions with HIV. Third, as the availability of COVID-19 vaccine and therapies, how these preventive and treatment methods are going to impact the outcome of SARS-CoV-2/HIV co-infection needs to be further illustrated. Fourth, the studies must focus on the pathological and immunological perspective of the interaction between HIV and COVID-19.

In conclusion, HIV infection remains an important risk factor for COVID-19 mortality. Therefore, it is highly recommended that PLWH should be protected from the COVID-19 pandemic through public health prevention and vaccines. On the other hand, overcoming current health system barriers (e.g., quarantine, social distancing, and community containment measurements) and securing continued ART supply as well as other HIV services is important for PLWH survival through the SARS-CoV-2 and HIV pandemics.

## Data Availability Statement

The original contributions presented in the study are included in the article/**Supplementary Material**. Further inquiries can be directed to the corresponding author.

## Author Contributions

WT and YW conceived the study idea; YW, YX, SH, WA, and YT conducted the searching, screening, and data extraction; YW, YX, FJ, and HT helped with the data analysis; WT, YW, and YX drafted the manuscript. All authors approved the manuscript for publication.

## Funding

This work was supported by the National Natural Science Foundation of China (81903371) and NIH (R34MH119963).

## Conflict of Interest

The authors declare that the research was conducted in the absence of any commercial or financial relationships that could be construed as a potential conflict of interest.

The handling editor declared a shared affiliation with the authors YX, FJ, WT at the time of review.

## Publisher’s Note

All claims expressed in this article are solely those of the authors and do not necessarily represent those of their affiliated organizations, or those of the publisher, the editors and the reviewers. Any product that may be evaluated in this article, or claim that may be made by its manufacturer, is not guaranteed or endorsed by the publisher.
