# Supplementary material for: Systematic Review and Meta-Analyses of The Interaction Between HIV Infection And COVID-19: Two Years’ Evidence Summary
Source: Front Immunol. 2022 May 10;13:864838. doi: 10.3389/fimmu.2022.864838 (PMC9128408; doi:10.3389/fimmu.2022.864838)

Supplemetary materials

[Supplementary table 1: Search Strategy 2](#_Toc94225360)

[Supplementary table 2. The basic characteristic of selected studies. 5](#_Toc94225361)

[Supplementary table 3. The patient-level sociodemographic and clinical information of selected studies. 7](#_Toc94225362)

[Supplementary table 4. The quality assessment of selected studies based on the STROBE-M. 9](#_Toc94225363)

[Supplementary figure 2.1: The forest plot of the sub-group analysis of the adjusted risk ratio of COVID-19 mortality, comparing the patients with and without HIV (HIC vs. LMIC). 10](#_Toc94225364)

[Supplementary figure 2.2: The forest plot of the sub-group analysis of the adjusted risk ratio of COVID-19 mortality, comparing the patients with and without HIV (only included inpatient cases vs. include both inpatient and outpatient cases). 11](#_Toc94225365)

[Supplementary figure 2.3: The forest plot of the sub-group analysis of the adjusted risk ratio of COVID-19 mortality, comparing the patients with and without HIV (multi-center studies vs. single-center studies). 12](#_Toc94225366)

[Supplementary figure 4: Funnel plot of the adjusted risk ratio of COVID-19 mortality, comparing the patients with and without HIV. 13](#_Toc94225367)

# Supplementary Table 1: Search Strategy

| **Search time frame: （2019/12/1-2021/12/1）** | |
| --- | --- |
| **PubMed** | |
| **Searching terms** | **N** |
| ((((((((((((((((((((((((((((((Case-Control Studies[Title/Abstract]) OR (Case-Control Studies[MeSH Major Topic])) OR (Cohort Studies[Title/Abstract])) OR (Cohort Studies[MeSH Major Topic])) OR (Cross-Sectional Studies[MeSH Major Topic])) OR (Cross-Sectional Studies[MeSH Major Topic])) OR (Controlled Before-After Studies[Title/Abstract])) OR (Controlled Before-After Studies[MeSH Major Topic])) OR (Historically Controlled Study[Title/Abstract])) OR (Historically Controlled Study[MeSH Major Topic])) OR (Observational Study[Title/Abstract])) OR (Retrospective Study[Title/Abstract])) OR (Retrospective Study[MeSH Major Topic])) OR (Prospective Study[Title/Abstract])) OR (Prospective Study[MeSH Major Topic])) OR (Case-Control Study[Title/Abstract])) OR (Case-Control Study[MeSH Major Topic])) OR (Cohort Study[Title/Abstract])) OR (Cohort Study[MeSH Major Topic])) OR (Cross-Sectional Study[Title/Abstract])) OR (Cross-Sectional Study[MeSH Major Topic])) OR (Controlled Before-After Study[Title/Abstract])) OR (Controlled Before-After Study[MeSH Major Topic])) OR (Historically Controlled Studies[Title/Abstract])) OR (Historically Controlled Studies[MeSH Major Topic])) OR (Observational Studies[Title/Abstract])) OR (Retrospective Studies[Title/Abstract])) OR (Retrospective Studies[MeSH Major Topic])) OR (Prospective Studies[Title/Abstract])) OR (Prospective Studies[MeSH Major Topic]))  AND (((((((((((((covid-19[Title/Abstract]) OR (covid-19[MeSH Major Topic])) OR (coronavirus[Title/Abstract])) OR (coronavirus[MeSH Major Topic])) OR (novel coronavirus[Title/Abstract])) OR (novel coronavirus[MeSH Major Topic])) OR (SARS-CoV*[Title/Abstract])) OR (SARS-CoV*[MeSH Major Topic])) OR (2019-nCoV[Title/Abstract])) OR (2019-nCoV[MeSH Major Topic])) OR (nCoV[Title/Abstract])) OR (nCoV[MeSH Major Topic]))  AND ((((((((HIV[Title/Abstract]) OR (HIV[MeSH Major Topic])) OR (human immunodeficiency virus*[Title/Abstract])) OR (human immunodeficiency virus*[MeSH Major Topic])) OR (AIDS[Title/Abstract])) OR (AIDS[MeSH Major Topic])) OR (acquired immunodeficiency syndrome[Title/Abstract])) OR (acquired immunodeficiency syndrome[MeSH Major Topic]))) | 181 |
| **EMBASE** |  |
| **Searching terms** | **N** |
| ('coronavirus disease 2019'/exp OR 'coronavirus disease 2019' OR '2019 novel coronavirus disease':ti,ab,kw OR '2019 novel coronavirus epidemic':ti,ab,kw OR '2019 novel coronavirus infection':ti,ab,kw OR '2019-ncov disease':ti,ab,kw OR '2019-ncov infection':ti,ab,kw OR 'covid':ti,ab,kw OR 'covid 19':ti,ab,kw OR 'covid 19 induced pneumonia':ti,ab,kw OR 'covid 2019':ti,ab,kw OR 'covid-10':ti,ab,kw OR 'covid-19':ti,ab,kw OR 'covid-19 induced pneumonia':ti,ab,kw OR 'covid-19 pneumonia':ti,ab,kw OR 'covid19':ti,ab,kw OR 'sars coronavirus 2 infection':ti,ab,kw OR 'sars coronavirus 2 pneumonia':ti,ab,kw OR 'sars-cov-2 disease':ti,ab,kw OR 'sars-cov-2 infection':ti,ab,kw OR 'sars-cov-2 pneumonia':ti,ab,kw OR 'sars-cov2 disease':ti,ab,kw OR 'sars-cov2 infection':ti,ab,kw OR 'sarscov2 disease':ti,ab,kw OR 'sarscov2 infection':ti,ab,kw OR 'wuhan coronavirus disease':ti,ab,kw OR 'wuhan coronavirus infection':ti,ab,kw OR 'coronavirus disease 2':ti,ab,kw OR 'coronavirus disease 2010':ti,ab,kw OR 'coronavirus disease 2019':ti,ab,kw OR 'coronavirus disease 2019 pneumonia':ti,ab,kw OR 'coronavirus disease-19':ti,ab,kw OR 'coronavirus infection 2019':ti,ab,kw OR 'ncov 2019 disease':ti,ab,kw OR 'ncov 2019 infection':ti,ab,kw OR 'new coronavirus pneumonia':ti,ab,kw OR 'novel coronavirus 2019 disease':ti,ab,kw OR 'novel coronavirus 2019 infection':ti,ab,kw OR 'novel coronavirus disease 2019':ti,ab,kw OR 'novel coronavirus infected pneumonia':ti,ab,kw OR 'novel coronavirus infection 2019':ti,ab,kw OR 'novel coronavirus pneumonia':ti,ab,kw OR 'paucisymptomatic coronavirus disease 2019':ti,ab,kw OR 'severe acute respiratory syndrome 2':ti,ab,kw OR 'severe acute respiratory syndrome 2 pneumonia':ti,ab,kw OR 'severe acute respiratory syndrome cov-2 infection':ti,ab,kw OR 'severe acute respiratory syndrome coronavirus 2 infection':ti,ab,kw OR 'severe acute respiratory syndrome coronavirus 2019 infection':ti,ab,kw) AND ('human immunodeficiency virus'/exp OR 'human immunodeficiency virus' OR 'aids virus':ti,ab,kw OR 'hiv':ti,ab,kw OR 'human immuno deficiency virus':ti,ab,kw OR 'human immunodeficiency virus':ti,ab,kw OR 'lav (aids)':ti,ab,kw OR 'lymphadenopathy associated virus':ti,ab,kw OR 'aids associated lentivirus':ti,ab,kw OR 'aids associated retrovirus':ti,ab,kw OR 'aids associated virus':ti,ab,kw OR 'aids related virus':ti,ab,kw OR 'immunodeficiency associated virus':ti,ab,kw OR 'immunodeficiency viruses, primate':ti,ab,kw OR 'lav':ti,ab,kw OR 'lymphadenopathy associated retrovirus':ti,ab,kw OR 'virus, lymphadenopathy associated':ti,ab,kw) AND ('case control study'/exp OR 'case control study' OR 'case control study':ti,ab,kw OR 'case-control studies':ti,ab,kw OR 'case-control study':ti,ab,kw OR 'control study, case':ti,ab,kw OR 'matched case control':ti,ab,kw OR 'matched case control studies':ti,ab,kw OR 'matched case control study':ti,ab,kw OR 'cohort analysis'/exp OR 'cohort analysis' OR 'analysis, cohort':ti,ab,kw OR 'cohort analysis':ti,ab,kw OR 'cohort fertility':ti,ab,kw OR 'cohort life cycle':ti,ab,kw OR 'cohort studies':ti,ab,kw OR 'cohort study':ti,ab,kw OR 'fertility, cohort':ti,ab,kw OR 'cross-sectional study'/exp OR 'cross-sectional study' OR 'cross-sectional design':ti,ab,kw OR 'cross-sectional research':ti,ab,kw OR 'cross-sectional studies':ti,ab,kw OR 'cross-sectional study':ti,ab,kw OR 'observational study'/exp OR 'observational study' OR 'non experimental studies':ti,ab,kw OR 'non experimental study':ti,ab,kw OR 'nonexperimental studies':ti,ab,kw OR 'nonexperimental study':ti,ab,kw OR 'observation studies':ti,ab,kw OR 'observation study':ti,ab,kw OR 'observational studies':ti,ab,kw OR 'observational studies as topic':ti,ab,kw OR 'observational study':ti,ab,kw OR 'observational study as topic':ti,ab,kw OR 'retrospective study'/exp OR 'retrospective study' OR 'ex post facto design':ti,ab,kw OR 'retrospective design':ti,ab,kw OR 'retrospective panel studies':ti,ab,kw OR 'retrospective panel study':ti,ab,kw OR 'retrospective studies':ti,ab,kw OR 'retrospective study':ti,ab,kw OR 'study, retrospective':ti,ab,kw OR 'prospective study'/exp OR 'prospective study' OR 'prospective method':ti,ab,kw OR 'prospective studies':ti,ab,kw OR 'prospective study':ti,ab,kw OR 'study, prospective':ti,ab,kw OR 'epidemiology'/exp OR 'epidemiology' OR 'controlled before after studies':ti,ab,kw OR 'controlled before and after studies':ti,ab,kw OR 'controlled before and after study':ti,ab,kw OR 'controlled before-after studies':ti,ab,kw OR 'historically controlled study':ti,ab,kw) AND   AND [1-12-2019]/sd NOT [1-12-2021]/sd | 1765 |
| **Cochrane** |  |
| **Searching terms** | **N** |
| (HIV):ti,ab,kw AND (covid):ti,ab,kw (Word variations have been searched) with Publication Year from 2019 to 2021 | 169 |
| **MEDLINE (Ovid)** |  |
| **Searching terms** | **N** |
| (exp COVID-19/ or exp SARS-CoV-2/ or exp Severe acute respiratory syndrome/ or exp Coronavirus/ or exp Coronavirus Infections/) AND (exp HIV/ or exp HIV Infections/ or exp Acquired Immunodeficiency Syndrome/) AND (exp Case-Control Studies/ or exp Cohort Studies/ or exp Cross-Sectional Studies/ or exp Controlled Before-After Studies/ or exp Historically Controlled Study/ or exp Observational Study/ or exp Retrospective Study/ or exp Prospective Study/) | 110 |

| Supplementary Table 2. The basic characteristic of selected studies. | | | | | | | | |
| --- | --- | --- | --- | --- | --- | --- | --- | --- |
| **First author** | **Year** | **Study country** | **Study period** | **Study design** | **Study settings** | **Multicenter study** | **Hospitalization cases only** | **Diagnosis method** |
| Western Cape Department of Health in collaboration with the National Institute for Communicable Diseases. (25) | 2021 | South Africa | Mar 1st, 2020 - June 9th, 2020 | Cohort study | data from the Western Cape Provincial Health Data Centre | Yes | No | PCR |
| Abdela et al. (26) | 2020 | Ethiopia | May 9th, 2020 - Sep 20th, 2020 | Retrospective cohort study | Hospital | No | Yes | PCR |
| Abraha et al. (27) | 2021 | Ethiopia | May 10th, 2020 - Oct 16th, 2020 | Retrospective cohort study | Hospital | No | No | PCR |
| Bennett et al. (28) | 2021 | United States | Starting from Dec 2020 | N.A. | Multicenter registry | Yes | No | PCR |
| Berenguer et al. (29) | 2021 | Spain | Apr 1st, 2020 - Sep 30th, 2020 | Prospective cohort | Spanish HIV Research Network Cohort | Yes | No | Antibody testing |
| Bergman et al. (30) | 2021 | Sweden | Until Sep 15th, 2020 | nationwide, registry-based study | National registry | Yes | No | N.A. |
| Bhaskaran et al. (31) | 2021 | United Kingdom | From Feb 1st, 2020 | Retrospective cohort study | OpenSAFELY platform | Yes | No | N.A. |
| Blanco et al. (32) | 2021 | Spain | Feb 26th, 2020 - Sep 21st, 2020 | Prospective case-cohort study | Nationwide multicenter prospective case-cohort study. | Yes | Yes | N.A. |
| Brown et al. (33) | 2021 | United Kingdom | Mar 2nd, 2020 – June 16th, 2020 | Retrospective cohort study | National HIV surveillance data | Yes | No | N.A. |
| Chanda et al. (34) | 2021 | Zambia | Mar 2020 – Dec 2020 | Cohort study | su | Yes | No | N.A. |
| Eybpoosh et al. (35) | 2021 | Iran | Feb 19th, 2020 - Apr 8th, 2020 | Retrospective cohort study | Hospital | Yes | Yes | PCR |
| Friedman et al. (36) | 2021 | United States | Apr 10th, 2020 - Sep 30th, 2020 | Cross-sectional study | Hospital | No | No | PCR |
| Gagliardini et al. (37) | 2021 | Italy | N.A. | N.A. | Hospital | Yes | Yes | PCR/nasopharyngeal swabs/serology |
| Ge et al. (38) | 2021 | Canada | Jan 15th, 2020 - Dec 31st, 2020 | Cohort study | Ontario Laboratories Information System (OLIS) | Yes | No | PCR |
| Geretti et al. (39) | 2021 | United Kingdom | Jan 17th, 2020 - June 18th, 2020 | Cohort study | ISARIC WHO CCP-UK | Yes | Yes | PCR |
| Hadi et al. (40) | 2020 | United States | N.A. | Cohort study | TriNETX, real-world cohort | Yes | No | N.A. |
| Inciarte et al. (41) | 2020 | Spain | Mar 1st, 2020 - May 10th, 2020 | Prospective cohort study | HIV cohort | No | No | PCR |
| Jassat et al. (42) | 2021 | South Africa | Mar 5th, 2020 - Mar 27th, 2021 | Cohort study | DATCOV, a national active hospital surveillance system | Yes | Yes | PCR |
| Karim et al. (43) | 2021 | South Africa | June 2020 - May 2021 | Cohort study | Hospital | Yes | Yes | PCR |
| Laracy et al. (44) | 2021 | United States | Mar 10th, 2020 - Jun 10th, 2020 | Retrospective cohort study | Hospital | Yes | No | PCR |
| Lee et al. (45) | 2021 | United Kingdom | Feb 1st, 2020 - May 31th,2020 | Retrospective cohort study | Hospital | Yes | Yes | PCR |
| Nagarakanti et al. (46) | 2021 | United States | Mar 2020 - Apr 2020 | Retrospective cohort study | Hospital | No | Yes | PCR |
| Nomah et al. (18) | 2021 | Spain | Mar 1th, 2020 - Dec 15th, 2020 | Retrospective cohort study | PISCIS cohort of people with HIV | Yes | No | PCR or antibody testing or antigen testing |
| Patel et al. (47) | 2021 | United States | Mar 10th, 2020 - May 11th, 2020 | Retrospective cohort study | Hospital | Yes | Yes | PCR |
| Sigel et al. (48) | 2020 | United States | Mar 12th, 2020 - Apr 23th 2020 | Matched case-control study | Hospital | Yes | Yes | PCR |
| Spinelli et al. (49) | 2021 | United States | Aug 1st, 2020 - Oct 31th, 2020 | Matched case-control study | Hospital | No | No | IgG test |
| Stoeckle et al. (50) | 2020 | United States | Mar 3th, 2020 - May 15th, 2020 | Retrospective cohort study | Hospital | Yes | Yes | N.A. |
| Tang et al. (51) | 2021 | United States | Mar 2020 - July 2020 | Retrospective cohort study | Hospital | Yes | No | N.A. |
| Tesoriero et al. (52) | 2021 | United States | Mar 1st, 2020 - June 15th, 2020 | Retrospective cohort study | HIV surveillance, COVID-19 laboratory-confirmed diagnoses, and hospitalization databases | Yes | No | PCR |
| Venturas et al. (53) | 2020 | South Africa | Mar 6th, 2020 - Sep 11th, 2020 | Prospective cohort study | Hospital | No | Yes | PCR |
| Yang et al. (54) | 2021 | United States | Jan 1st, 2020 - May 8th, 2021 | Cohort study | National COVID Cohort Collaborative | Yes | No | Positive SARS-CoV-2 laboratory tests |
| Yendewa et al. (55) | 2021 | United States | Jan 1th,2020 - Dec 1th, 2020 | Retrospective cohort study | Hospital | Yes | No | PCR or serology testing |

| Supplementary table 3. The patient-level sociodemographic and clinical information of selected studies. | | | | | | | | | | | | | | | | | | | |
| --- | --- | --- | --- | --- | --- | --- | --- | --- | --- | --- | --- | --- | --- | --- | --- | --- | --- | --- | --- |
| **Study** | **Year** | **Male among HIV+** | **Male among HIV-** | **The average age among HIV+** | **The average age among HIV-** | **On ART** | **Viral suppressed** | **Any comorbidity** | | **Hypertension** | | **Diabetes** | | **Obesity** | | **COPD or Athema** | | **Cardiovascular diseases** | |
|  |  |  |  |  |  |  |  | **Among HIV+ (%)** | **Among HIV- (%)** | **Among HIV+ (%)** | **Among HIV- (%)** | **Among HIV+ (%)** | **Among HIV- (%)** | **Among HIV+ (%)** | **Among HIV- (%)** | **Among HIV+ (%)** | **Among HIV- (%)** | **Among HIV+ (%)** | **Among HIV- (%)** |
| Western Cape Department of Health in collaboration with the National Institute for Communicable Diseases. (25) | 2021 | 22% | 34% | ^-^ | ^-^ | ^-^ | 31% | ^-^ | ^-^ | 12% | 17% | 4% | 8% | ^-^ | ^-^ | 4% | 6% | ^-^ | ^-^ |
| Abdela et al. (26) | 2020 | ^-^ | ^-^ | ^-^ | ^-^ | ^-^ | ^-^ | ^-^ | ^-^ | ^-^ | ^-^ | ^-^ | ^-^ | ^-^ | ^-^ | ^-^ | ^-^ | ^-^ | ^-^ |
| Abraha et al. (27) | 2021 | ^-^ | ^-^ | ^-^ | ^-^ | ^-^ | ^-^ | ^-^ | ^-^ | ^-^ | ^-^ | ^-^ | ^-^ | ^-^ | ^-^ | ^-^ | ^-^ | ^-^ | ^-^ |
| Bennett et al. (28) | 2021 | 75% | 49% | ^-^ | ^-^ | ^-^ | ^-^ | ^-^ | ^-^ | 39% | 41% | 24% | 23% | ^-^ | ^-^ | 10% | 9% | 8% | 9% |
| Berenguer et al. (29) | 2021 | 88% | ^-^ | ^-^ | ^-^ | 93% | 88% | ^-^ | ^-^ | 38% | ^-^ | 2% | ^-^ | ^-^ | ^-^ | ^-^ | ^-^ | 3% | ^-^ |
| Bergman et al. (30) | 2021 | ^-^ | ^-^ | ^-^ | ^-^ | ^-^ | ^-^ | ^-^ | ^-^ | ^-^ | ^-^ | ^-^ | ^-^ | ^-^ | ^-^ | ^-^ | ^-^ | ^-^ | ^-^ |
| Bhaskaran et al. (31) | 2021 | 65% | 50% | ^-^ | ^-^ | ^-^ | ^-^ | ^-^ | ^-^ | 19% | 21% | 10% | 10% | ^-^ | ^-^ | ^-^ | ^-^ | 5% | 7% |
| Blanco et al. (32) | 2021 | ^-^ | ^-^ | ^-^ | ^-^ | ^-^ | 90% | ^-^ | ^-^ | ^-^ | ^-^ | ^-^ | ^-^ | ^-^ | ^-^ | ^-^ | ^-^ | ^-^ | ^-^ |
| Brown et al. (33) | 2021 | 69% | 49% |  |  | 99% | 91% | 90% | ^-^ | 69% | ^-^ | 48% | ^-^ | 49% | ^-^ | 10% | ^-^ | 69% | ^-^ |
| Chanda et al. (34) | 2021 | 52% | 59% | 46.4 | 48.9 | 89% | 86% | 44% | 52% | 26% | 36% | 15% | 14% | ^-^ | ^-^ | ^-^ | ^-^ | ^-^ | ^-^ |
| Eybpoosh et al. (35) | 2021 | ^-^ | ^-^ | ^-^ | ^-^ | ^-^ | ^-^ | ^-^ | ^-^ | ^-^ | ^-^ | ^-^ | ^-^ | ^-^ | ^-^ | ^-^ | ^-^ | ^-^ | ^-^ |
| Friedman et al. (36) | 2021 | 71% | 43% | 47 | 58 | 77% | ^-^ | 64% | ^-^ | 48% | ^-^ | ^-^ | ^-^ | 11% | ^-^ | 28% | ^-^ | 14% | ^-^ |
| Gagliardini et al. (37) | 2021 | ^-^ | ^-^ | 56 | 62 | ^-^ | 94% | ^-^ | ^-^ | ^-^ | ^-^ | ^-^ | ^-^ | ^-^ | ^-^ | ^-^ | ^-^ | ^-^ | ^-^ |
| Ge et al. (38) | 2021 | ^-^ | ^-^ | ^-^ | ^-^ | ^-^ | ^-^ | ^-^ | ^-^ | ^-^ | ^-^ | ^-^ | ^-^ | ^-^ | ^-^ | ^-^ | ^-^ | ^-^ | ^-^ |
| Geretti et al. (39) | 2021 | 66% | 57% | 54 | 54 | 92% | ^-^ | 75% | 79% | ^-^ | ^-^ | 21% | 25% | 17% | 11% | 10% | 14% | 17% | 32% |
| Hadi et al. (40) | 2020 | 71% | 45% | 48.18 | 48.8 | 70% | ^-^ | ^-^ | ^-^ | 46% | 28% | 22% | 15% | 25% | 21% | ^-^ | ^-^ | 14% | 8% |
| Inciarte et al. (41) | 2020 | 83% | 0% | 47 | ^-^ | 96% | 96% | ^-^ | ^-^ | ^-^ | ^-^ | ^-^ | ^-^ | ^-^ | ^-^ | ^-^ | ^-^ | ^-^ | ^-^ |
| Jassat et al. (42) | 2021 | 35% | 46% | ^-^ | ^-^ | 93% | 74% | ^-^ | ^-^ | 28% | 34% | 19% | 25% | ^-^ | ^-^ | 7% | 6% | 2% | 2% |
| Karim et al. (43) | 2021 | 37% | 34% | 41 | 49 | ^-^ | 88% | ^-^ | ^-^ | 16% | 29% | 11% | 22% | 29% | 47% | ^-^ | ^-^ | ^-^ | ^-^ |
| Laracy et al. (44) | 2021 | 71% | 71% | 59 | 58.2 | 99% | 47% | ^-^ | ^-^ | 63% | 43% | 37% | 29% | ^-^ | ^-^ | ^-^ | ^-^ | ^-^ | ^-^ |
| Lee et al. (45) | 2021 | 62% | 63% | 57 | 56 | 93% | 97% | ^-^ | ^-^ | 51% | 41% | 26% | 29% | ^-^ | ^-^ | 4% | 13% | 18% | 12% |
| Nagarakanti et al. (46) | 2021 | 61% | 50% | 59 | 62 | 91% | 70% | ^-^ | ^-^ | 65% | 70% | 30% | 47% | ^-^ | ^-^ | 4% | 9% | 9% | 22% |
| Nomah et al. (18) | 2021 | 82% | 0% | 47.6 | ^-^ | 94% | 83% | 66% | 0% | 21% | 0% | 6% | 0% | 8% | 0% | ^-^ | ^-^ | - | 0% |
| Patel et al. (47) | 2021 | 55% | 53% | 63 | 65 | 90% | 81% | ^-^ | ^-^ | 73% | 68% | 43% | 45% | 35% | 35% | ^-^ | ^-^ | 18% | 14% |
| Sigel et al. (48) | 2020 | 75% | 76% | 61 | 60 | 100% | ^-^ | ^-^ | ^-^ | 38% | 33% | 27% | 29% | 10% | 8% | 9% | 2% | 7% | 11% |
| Spinelli et al. (49) | 2021 | 82% | 49% | 54 | 57 | ^-^ | ^-^ | ^-^ | ^-^ | ^-^ | ^-^ | 22% | 54% | ^-^ | ^-^ | 11% | 9% | 37% | 56% |
| Stoeckle et al. (50) | 2020 | 80% | 80% | 60.5 | 60.5 | 97% | ^-^ | ^-^ | ^-^ | 40% | 53% | 27% | 33% | ^-^ | ^-^ | 12% | 6% | 3% | 7% |
| Tang et al. (51) | 2021 | 85% | 39% | ^-^ | ^-^ | ^-^ | ^-^ | ^-^ | ^-^ | ^-^ | ^-^ | ^-^ | ^-^ | ^-^ | ^-^ | ^-^ | ^-^ | ^-^ | ^-^ |
| Tesoriero et al. (52) | 2021 | 72% | 48% | ^-^ | ^-^ | ^-^ | ^-^ | ^-^ | ^-^ | ^-^ | ^-^ | ^-^ | ^-^ | ^-^ | ^-^ | ^-^ | ^-^ | ^-^ | ^-^ |
| Venturas et al. (53) | 2020 | 50% | 54% | 45 | 52.5 | ^-^ | ^-^ | 67% | 72% | ^-^ | ^-^ | 17% | 27% | 12% | 25% | 6% | 4% | 33% | 41% |
| Yang et al. (54) | 2021 | 73% | 45% | 49 | 47 | ^-^ | 82% | ^-^ | ^-^ | ^-^ | ^-^ | 23% | 16% | 19% | 15% | ^-^ | ^-^ | 38% | 24.19% |
| Yendewa et al. (55) | 2021 | 69% | 44% | 43.34 | 46.48 | 83% | 38% | ^-^ | ^-^ | ^-^ | ^-^ | 22% | 13% | 25% | 15% | 14% | 8% | 60% | 33% |

| Supplementary Table 4. The quality assessment of selected studies based on the STROBE-M. | | | |
| --- | --- | --- | --- |
| **Study** | **Score** | **Category** | **Rank** |
| Western Cape Department of Health in collaboration with the National Institute for Communicable Diseases. (25) | 65.5 | 3 | Fair |
| Abdela et al. (26) | 56.0 | 3 | Fair |
| Abraha et al. (27) | 64.3 | 3 | Fair |
| Bennett et al. (28) | 61.9 | 3 | Fair |
| Berenguer et al. (29) | 61.9 | 3 | Fair |
| Bergman et al. (30) | 65.5 | 3 | Fair |
| Bhaskaran et al. (31) | 81.0 | 2 | Good |
| Blanco et al. (32) | 40.5 | 4 | Poor |
| Brown et al. (33) | 76.2 | 2 | Good |
| Chanda et al. (34) | 70.2 | 2 | Good |
| Eybpoosh et al. (35) | 73.8 | 2 | Good |
| Friedman et al. (36) | 64.3 | 3 | Fair |
| Gagliardini et al. (37) | 44.0 | 4 | Poor |
| Ge et al. (38) | 82.1 | 2 | Good |
| Laracy et al. (39) | 63.1 | 3 | Fair |
| Lee et al. (40) | 71.4 | 2 | Good |
| Nagarakanti et al. (41) | 63.1 | 3 | Fair |
| Nomah et al. (18) | 71.4 | 2 | Good |
| Patel et al. (42) | 81.0 | 2 | Good |
| Sigel et al. (43) | 72.6 | 2 | Good |
| Spinelli et al. (44) | 79.8 | 2 | Good |
| Stoeckle et al. (45) | 72.6 | 2 | Good |
| Tang et al. (46) | 61.9 | 3 | Fair |
| Tesoriero et al. (47) | 81.0 | 2 | Good |
| Venturas et al. (48) | 78.6 | 2 | Good |
| Yendewa et al. (49) | 67.9 | 3 | Fair |
| Geretti et al. (50) | 77.4 | 2 | Good |
| Hadi et al. (51) | 57.1 | 3 | Fair |
| Inciarte et al. (52) | 56.0 | 3 | Fair |
| Jassat et al. (53) | 86.9 | 1 | Excellent |
| Karim et al. (54) | 73.8 | 2 | Good |
| Yang et al. (55) | 88.1 | 1 | Excellent |

# Supplementary Figure 2.1: The forest plot of the sub-group analysis of the adjusted risk ratio of COVID-19 mortality, comparing the patients with and without HIV (HIC vs. LMIC).


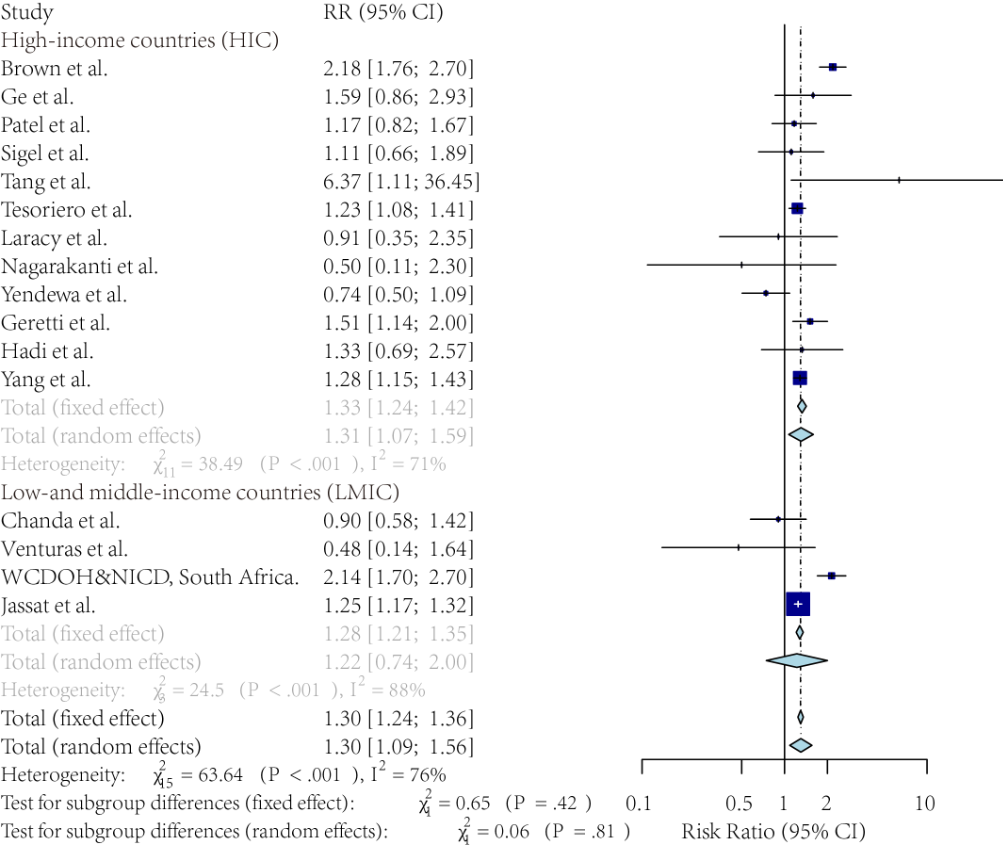


# Supplementary Figure 2.2: The forest plot of the sub-group analysis of the adjusted risk ratio of COVID-19 mortality, comparing the patients with and without HIV (only included inpatient cases vs. both inpatient and outpatient cases).


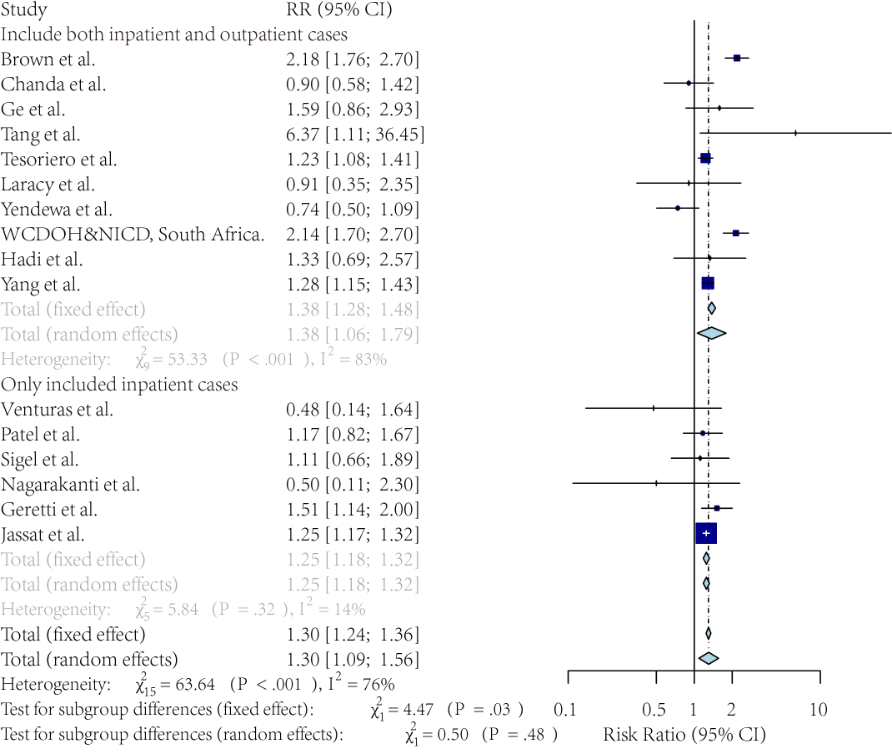


# Supplementary Figure 2.3: The forest plot of the sub-group analysis of the adjusted risk ratio of COVID-19 mortality, comparing the patients with and without HIV (multicenter studies vs. single-center studies).


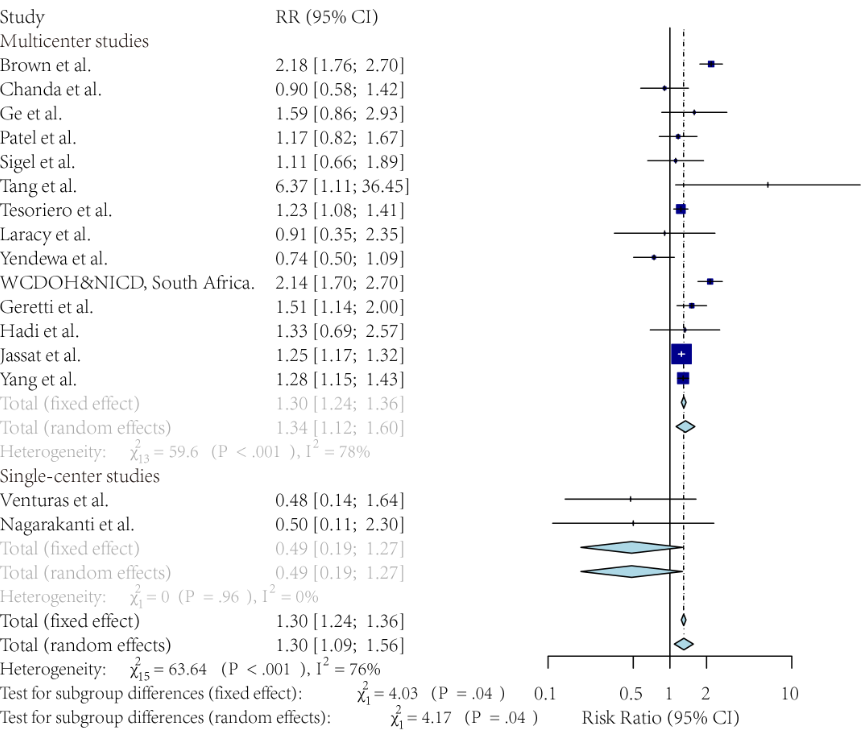


# Supplementary Figure 4: Funnel plot of the adjusted risk ratio of COVID-19 mortality, comparing the patients with and without HIV.


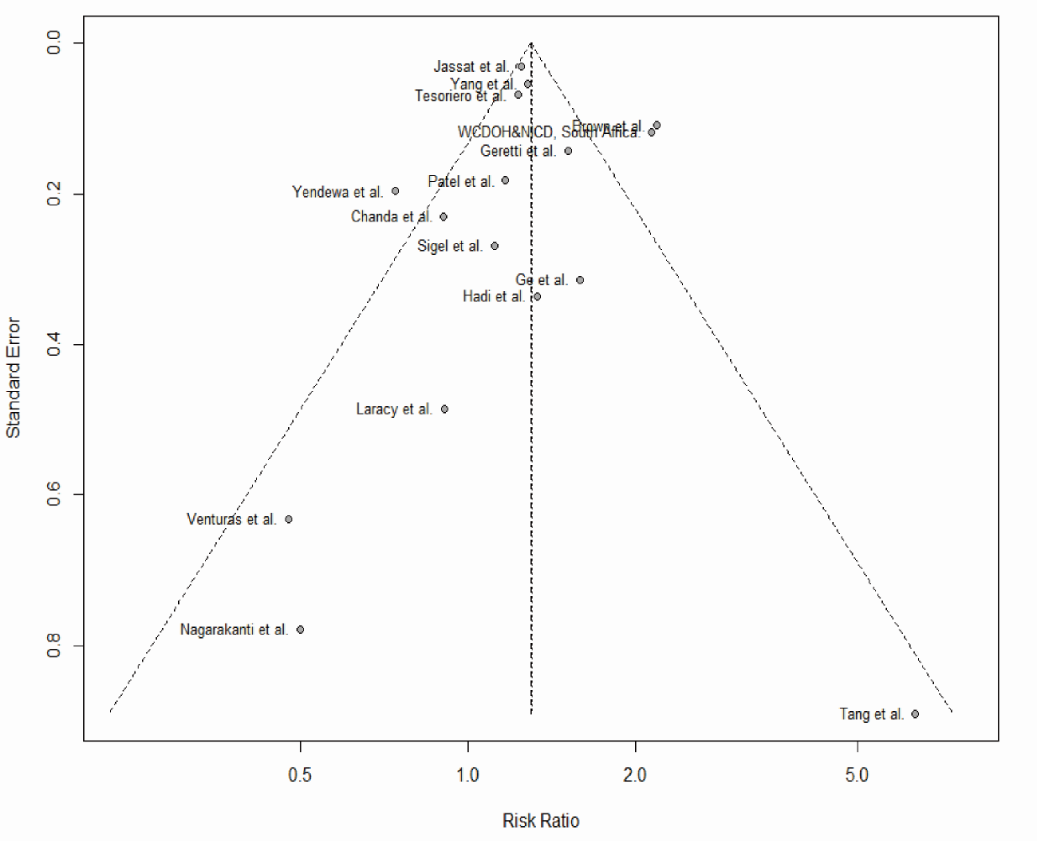

Supplement: Supplementary file 1 [file DataSheet_1.docx]
